# Effect of Wort Boiling on Volatiles Formation and Sensory Properties of Mead

**DOI:** 10.3390/molecules27030710

**Published:** 2022-01-21

**Authors:** Małgorzata Starowicz, Michael Granvogl

**Affiliations:** 1 Department of Chemistry and Biodynamics of Food, Institute of Animal Reproduction and Food Research, Polish Academy of Sciences, Tuwima Street 10, 10-748 Olsztyn, Poland; 2 Fachgebiet für Lebensmittelchemie und Analytische Chemie (170a), Institut für Lebensmittelchemie, Fakultät Naturwissenschaften, Universität Hohenheim, Garbenstraße 28, D-70599 Stuttgart, Germany; michael.granvogl@uni-hohenheim.de

**Keywords:** mead, alcoholic beverages, Maillard reaction, wort boiling, aroma compounds, sensory profile

## Abstract

Mead is an alcoholic beverage based on bee honey, which can be prepared in different variations such as modified honey-water compositions, the addition of spices, and the use of different yeast strains. Moreover, the technological process of mead production such as the step of wort preparation (with or without boiling of wort before fermentation) can be modified. All these factors might have a significant impact on the formation of aroma-active compounds, and therefore, sensory acceptance by consumers. High vacuum distillation, using the so-called solvent assisted flavor evaporation (SAFE) technique, or headspace-solid phase microextraction (HS-SPME) were applied for the isolation of the odorants. A sensory profile was used to monitor the changes in the aroma of the mead samples. Twenty-eight aroma-active compounds were detected during aroma extract dilution analysis (AEDA) based on gas chromatography-olfactometry (GC-O) and were finally identified by gas chromatography-mass spectrometry (GC-MS) using authentic reference compounds, including methyl propanoate, methyl 3-(methylthio)propanoate, and methional, all of them were identified for the first time in mead. Compounds with high flavor dilution (FD) factors were quantitated via stable isotope dilution analysis (SIDA) and revealed ethyl acetate (16.4 mg/L) to be the most abundant volatile compound, increasing to 57 mg/L after wort boiling, followed by ethyl hexanoate (both 1.2 mg/L). Furthermore, key aroma compounds were esters such as ethyl hexanoate, ethyl octanoate, and ethyl 3-methylbutanoate. The sensory panel evaluated ethanolic, honey-like, clove-like, sweet, and fruity notes as the main aroma descriptors of mead. The significant change in sensory evaluation was noted in the sweet odor of the heat-treated mead.

## 1. Introduction

Mead is an alcoholic beverage with an alcohol content between 8 and 18%vol. It is traditionally produced in countries of Middle-Eastern Europe and is available in many variations. Due to various possible additives such as fruit juices, spices, and herbs used during mead production, different mead types known as *pyments*, *cysers*, *melomels*, and *metheglin* exist [1]. In Poland, mead is divided into groups by taking into consideration the ratio of honey to water, such as: ‘czwórniak’ (1:3, *v*:*v*; honey to water), ‘trójniak’ (1:2), ‘dwójniak’ (1:1), and ‘półtorak’ (1:0.5) [2,3,4]. Therefore, the classification of mead ranges from the noblest quality with the highest amount of honey and the longest fermentation time (‘półtorak’) to mead with lower quality, consisting of only one part of honey to three parts of water (‘czwórniak’). Of course, this fact is directly reflected by the respective price of the final product.

Mead is produced by few breweries, and it is also a popular product among home manufacturers. The process starts with the dilution of honey with an appropriate volume of water, followed by the fermentation of the wort with *Saccharomyces cerevisiae* [5]. Finally, mead is siphoned, bottled, pasteurized, and matured in bottles. It is already known that in fermented alcoholic beverages, the main volatile compounds are formed during the fermentation process [6,7,8,9]. However, it is important to note that all types of honey do not guarantee high sensory acceptance of the corresponding mead. The type, origin, and quality of honey play an important role in the overall sensorial properties of this alcoholic beverage [10,11]. Furthermore, the yeast strain used and the fermentation conditions significantly influence the formation of volatile compounds/odorants, particularly aldehydes, acids, higher alcohols, and esters. According to Sroka and Tuszyński [12], short-chain fatty acids enhanced the flavor of mead, and they are also precursors of volatile esters that showed high odor activity values (OAVs; defined as the ratio of the odorant concentration to its odor threshold) indicating their high potential to contribute to the overall aroma of mead. The volatile compounds formed in mead, including esters, greatly impact their sensory acceptability. Pino and Fajardo [13] reported that 11 compounds, mostly esters, were the most potent odorants in Cuban honey spirits using gas chromatography-olfactometry (GC-O) and gas chromatography-mass spectrometry (GC-MS). In buckwheat mead, 2-phenylethanol and a wide range of esters, including isoamyl acetate (banana-like aroma), were found to be key aroma compounds exhibiting high OAVs [14]. Besides aroma-active compounds, Gomes et al. [15] noted that the sugar content is one of the main factors influencing the acceptability of mead. The authors revealed that “sweet” mead was more preferred by consumers compared to “dry” ones, whereas the alcohol content did not influence the sensory acceptance.

Heat treatment of wort boiling can also improve the product quality, while only gentle boiling can increase the fermentation stability with a simultaneous increase of the desired aroma. Otherwise, intense heating can lead to an undesired overall mead aroma [16,17]. Bednarek and Szwengiel [18] recently noted that the quality of mead decreased after wort boiling and that heated mead is a good source of polyphenols only after fruit juice addition. The long period of heat treatment required for honey pasteurization was also associated with the formation of an off-flavor, described as rubbery and resin-like [14]. Further, it was reported that heat treatment stimulated the formation of 4-methylphenol, which possesses an unpleasant phenolic odor [14]. In this context, heating might have a negative impact on the organoleptic properties of the final product. Thus, aroma development in mead should be taken into consideration during fermentation, but also during the heat treatment of wort leading to the formation of Maillard reaction products. According to the literature previously collected in a review article about mead [19], the influence of wort boiling on aroma formation has not been studied in detail up to now. Consequently, specific compounds generated by overheating of mead have not been established as “thermal” markers. Moreover, the impact of wort boiling on the overall aroma profile of mead has not yet been reported.

Thus, the present study aimed at elucidating the differences in the key aroma compounds of two mead samples varying in their overall aroma that were either prepared without wort boiling (‘trójniak’—T) or with wort boiling (‘trójniak sycony’—TS) using the molecular sensory science concept (sensomics concept) including (1) the identification of the most important odorants on the basis of comparative aroma extract dilution analysis (cAEDA) based on GC-O and GC-MS, (2) the quantitation by means of stable isotope dilution assays (SIDAs) using GC-MS in combination with solid phase microextraction (SPME), and (3) the calculation of OAVs using orthonasal odor thresholds determined in an ethanol/water matrix.

## 2. Results and Discussion

### 2.1. Identification of Aroma-Active Compounds in Mead

**Figure 1:**
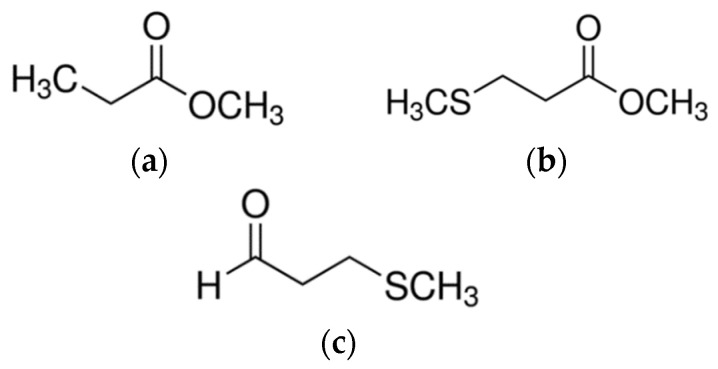
Aroma compounds determined for the first time in mead using GC-O technique: (**a**) methyl propanoate, (**b**) methyl 3-(methylthio)propanoate, and (**c**) methional.

cAEDA, based on GC-O, was applied to characterize the main differences in the aroma profiles of mead with and without wort boiling. Therefore, the odorants were identified according to their odor quality and intensity, retention indices on two columns of different polarities (DB-FFAP and DB-5), and mass spectra in the EI and CI mode in comparison to authentic reference compounds. The obtained odor qualities detected at the sniffing port during AEDA were compared to the data available in an in-house database containing over 1000 odorants. Some of the odorants found have previously been reported in mead and other fermented alcoholic beverages [13,14,20]; however, some of the aroma compounds detected in the present study were found for the first time in mead, e.g., methyl propanoate, methyl 3-(methylthio)propanoate, and methional (Figure 1).

Altogether, 28 aroma-active regions were determined in the flavor dilution (FD) factor range between 32 and 2048 (Table 1). In ‘T’ mead, the highest FD factor of 1024 was determined for **1** with a fruity and blueberry-like aroma and **22** with a clove-like aroma, followed by **5** (fruity, pineapple-like), **7** (fruity, green), **19** (flowery, honey-like), and **27** (beeswax-like, honey-like) (all FD factor of 512) and **23** (seasoning-like, spicy) and **24** (peach-like) (both FD factor of 256). In ‘TS’ mead, the highest FD factor of 2048 was analyzed for **5** (fruity, pineapple-like) and **22** (clove-like), followed by **19** (flowery, honey-like), **27** (beeswax-like, honey-like) (both FD factor of 1024), **1** (fruity, blueberry-like), **7** (fruity, green), **28** (vanilla-like, sweet) (all FD factor of 512), and **13** (etherical) (FD factor of 256).

Aside from some similarities in the analyzed samples, such as the determination of compounds **3** (banana-like, fruity), **9** (cooked potato-like), and **16** (sweaty), many more differences were identified. Compounds **6** (citrus-like, green), **11** (cheese-like, sweaty), **15** (fruity, honey-like), **18** (coconut-like), and **25** (wax-like) were only detected in ‘T’, whereas compounds **8** (vinegar-like), **12** (popcorn-like, roasty), **17** (cinnamon-like, fruity), and **21** (carrot-like, musty) were only determined in ‘TS’. Further, **5** (fruity, pineapple-like), **10** (cabbage-like, earthy), **13** (etherical), **14** (aniseed-like, hay-like, fishy), **19** (flowery, honey-like), **20** (caramel-like, sweet), **22** (clove-like), **26** (woodruff-like, almond paste-like), **27** (beeswax-like, honey-like), and **28** (vanilla-like, sweet) were found with higher FD factors in ‘TS’ compared to ‘T’. Otherwise, compounds **1** (fruity, blueberry-like), **23** (seasoning-like, spicy), and **24** (peach-like) were present with higher FD factors in ‘T’ than in ‘TS’.

The results obtained for ‘T’ mead were in agreement with a study performed by Pino and Fajardo [13], who reported FD values of 1024 for ethyl 3-methylbutanoate and of 512 for ethyl hexanoate. According to Pereira et al. [21], ethyl acetate, hexanoic acid, and octanoic acid led to an off-flavor in mead. Hexanoic acid was identified both in ‘T’ and ‘TS’ samples; moreover, its FD factor was the same. While wort boiling did not influence the occurrence of hexanoic acid, octanoic acid with its characteristic carrot-like and musty smell only appeared in ‘TS’ mead. Likewise, acetic acid, 2-acetylpyrazine, and ethyl 3-phenylpropanoate were only detected in ‘TS’. Acetic acid can be formed from ethanol during the fermentation process, but it can also derive from honey [22]. The appearance of 2-acetylpyrazine in ‘TS’ could be defined as a marker for heat treatment of mead wort. This pyrazine with a characteristic popcorn-like and roasty odor note is a Maillard-reaction product and is formed in foods treated at temperatures >100 °C [23]. The FD factor of methyl 3-(methylthio)propanoate with an unpleasant cabbage-like and earthy odor also increased after wort boiling. Moreover, esters such as ethyl hexanoate, diethyl succinate, and ethyl 3-phenylpropanoate were also previously determined in cherry wines [24].

**Table 1:** Volatile compounds identified in mead ‘trójniak’ (T) and ‘trójniak sycony’ (TS) during aroma extraction dilution analysis.

No. ^1^	Compound ^2^	Odor Quality ^3^	RIs ^4^		FD Factors ^5^	
			DB-FFAP	DB-5	T	TS
1	ethyl 3-methylbutanoate	fruity, blueberry-like	1013	775	1024	512
2	2-methyl-1-propanol	malty	1101	640	64	64
3	3-methylbutyl acetate	banana-like, fruity	1170	878	32	32
4	1,8-cineol	eucalyptus-like	1193	1036	32	128
5	ethyl hexanoate	fruity, pineapple-like	1207	739	512	2048
6	octanal	citrus-like, green	1280	1003	32	nd ^6^
7	ethyl octanoate	fruity, green	1425	1200	512	512
8	acetic acid	vinegar-like	1443	612	nd ^6^	32
9	methional	cooked potato-like	1448	905	64	64
10	methyl 3-(methylthio)propanoate	cabbage-like, earthy	1517	1034	<32	32
11	methyl propanoate	cheese-like, sweaty ^7^	1558	789	32	nd ^6^
12	2-acetylpyrazine	popcorn-like, roasty	1609	1024	nd ^6^	64
13	diethyl succinate	etherical ^7^	1665	996	32	256
14	3-methylnonane-2,4-dione	aniseed-like, hay-like, fishy	1708	1251	<32	128
15	pentyl acetate	fruity, honey-like ^7^	1814	1256	128	nd ^6^
16	hexanoic acid	sweaty	1836	1018	32	32
17	ethyl 3-phenylpropanoate	cinnamon-like, fruity	1867	1418	nd ^6^	32
18	*trans*-whisky lactone	coconut-like	1876	1303	128	nd ^6^
19	2-phenylethanol	flowery, honey-like	1905	1160	512	1024
20	4-hydroxy-2,5-dimethyl-3(2*H*)-furanone (furaneol^®^)	caramel-like, sweet	2030	1071	<32	64
21	octanoic acid	carrot-like, musty	2052	1279	nd ^6^	32
22	4-allyl-2-methoxyphenol	clove-like	2164	1359	1024	2048
23	3-hydroxy-4,5-dimethyl-2(5*H*)-furanone (sotolon)	seasoning-like, spicy	2195	1108	256	32
24	γ-decalactone	peach-like	2369	1680	256	32
25	dodecanoic acid	wax-like	2455	2169	128	nd ^6^
26	coumarin	woodruff-like, almond paste-like ^7^	2461	1442	<32	64
27	phenylacetic acid	beeswax-like, honey-like	2555	1261	512	1024
28	vanillin	vanilla-like, sweet	2569	1406	<32	512

^1^ Odorants were consecutively numbered according to their retention indices on DB-FFAP capillary column. ^2^ Odorants were identified by comparing their odor qualities and intensities, retention indices on capillary columns DB-FFAP and DB-5, and mass spectra (EI and CI mode) to data of authentic reference compounds. ^3^ Odor quality perceived at the sniffing port during GC-O. ^4^ Retention indices, calculated from the retention time of the compound and the retention times of adjacent *n*-alkanes by linear interpolation. ^5^ Flavor dilution factor: highest dilution of the concentrated SAFE distillate in which the odorant was detected during GC-O (DB-FFAP capillary column) for the last time. ^6^ Not detected. ^7^ Odor quality according to database from www.pherobase.com (accessed on 9 February 2021) [25].

### 2.2. Quantitation of Odorants via HS-SPME-HRGC-MS Using SIDA and Calculation of Their OAVs

The HS-SPME-HRGC-MS method allows for the analysis of volatile compounds in food samples [26], especially by the use of stable isotope dilution assays (SIDAs). Therefore, this method was applied to ‘T’ and ‘TS’ samples to quantitate the most important aroma-active compounds by integrating the signals of selected ions of each analyte and the corresponding stable isotopically labeled internal standard in combination with the respective response factor, which are presented in Table 2.

Previous studies characterized the volatile components of mead and determined the most abundant volatile alcohols, esters, carbonyls, phenols, fatty acids, and terpenes [11,12,13,14,27,28]. As a result, alcohols were quantitatively demonstrated to be the largest group of volatile compounds in mead, while esters were the second largest group [27,28]. In this study, ethyl acetate showed by far the highest concentration in ‘T’, followed by ethyl hexanoate, 1-pentanol, 2-phenylethanol, phenylacetic acid, 2-methyl-1-propanol, and ethyl decanoate. Lower amounts were found for esters like diethyl succinate, ethyl octanoate, and ethyl 3-methylbutanoate as well as for 4-allyl-2-methoxyphenol and 1,8-cineol (Table 3).

Additionally, in ‘TS’, ethyl acetate was determined with the highest concentration (3.5-times higher than in ‘T’), followed by ethyl hexanoate, 2-methyl-1-propanol, 1-pentanol, and phenylacetic acid. Moreover, the total amount of aroma compounds determined in ‘TS’ was almost three times higher compared to that determined in ‘T’ (63.96 and 23.01 mg L^−1^, respectively). This finding was in agreement with a study performed by Wintersteen et al. [14], who found that the total amount of volatiles in buckwheat mead was significantly higher in the high heat product. Significant differences in compound concentrations between ‘T’ and ‘TS’ samples were found for ethyl acetate, 4-allyl-2-methoxyphenol, 1,8-cineol, ethyl 3-methylbutanoate, 2-methyl-1-propanol, and 2-phenylethanol. The difference in concentrations of 4-allyl-2-methoxyphenol and 1,8-cineol, also known as eugenol and eucalyptol, could be explained by different amounts of spices added during mead preparation.

While 3.5- and 1.5-times higher amounts of ethyl acetate and 2-methyl-1-propanol were analyzed in ‘TS’, 1.5-times higher concentrations of ethyl 3-methylbutanoate and 2-phenylethanol were found in ‘T’. Šmogrovicová et al. [27] found that ethyl acetate was the main component of mead volatiles. The concentration of ethyl acetate in ‘T’ was similar to the amounts of ethyl acetate found in South African mead, whereas the concentration in ‘TS’ was in the range reported in Slovak ones. Moreover, almost 85% and 94%, respectively, of the overall concentration of aroma-active compounds in ‘T’ and ‘TS’ samples consisted of esters. Many of these esters had high OAVs (Table 4), which means that they have a high impact on the overall aroma of mead and positively influence mead acceptance [12].

The calculation of OAVs indicated ethyl hexanoate, ethyl octanoate, ethyl 3-methylbutanoate, and 1,8-cineol to have the highest OAVs in mead (Table 4). Further odorants with OAVs > 1 include 2-methyl-1-propanol, 4-allyl-2-methoxyphenol, ethyl acetate, and ethyl decanoate. In contrast, for diethyl succinate, 1-pentanol, phenylacetic acid, and 2-phenylethanol OAVs < 1 were calculated. Wintersteen et al. [14] noted that 2-phenylethanol was the key aroma compound in mead made from buckwheat honey, while a wide range of esters was also identified, revealing high OAVs. In the actual study, esters such as ethyl hexanoate, ethyl octanoate, and ethyl 3-methylbutanoate were found to be the dominant ones but not 2-phenylethanol with an OAV < 1 in both samples.

### 2.3. Aroma Profiles of Mead ‘Trójniak’ (T) and ‘Trójniak Sycony’ (TS)

**Figure 2:**
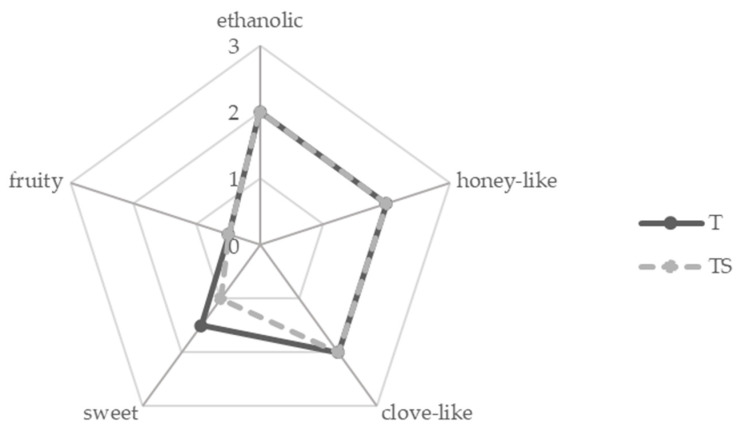
Aroma profiles of mead T (solid, dark grey line) and TS (broken, light grey line).

The sensory characteristic is an important criterion for product acceptability by consumers. Until now, only few studies reported on the sensory evaluation of various types of mead with regard to different honey type [11], fining agents [4], pollen addition [29], fruit/herbal additives [30], or mead fermented at different temperatures and with nutrition addition [31]. In each study, a sensory analysis of mead distinguished the samples due to differences in the additives used. However, only Kime et al. [32] evaluated the sensory profile of mead after wort boiling. Therefore, an aroma profile analysis (APA) was performed in this study to evaluate the overall aroma of mead ‘trójniak’ (T) and ‘trójniak sycony’ (TS) using the following aroma descriptors: ethanolic, honey-like, clove-like, sweet, and fruity. The ‘T’ and ‘TS’ aroma was described mostly as ethanolic (2.0) and honey-like (2.0; associated with phenylacetic acid) (Figure 2). These aroma attributes were in agreement with previous studies conducted by Li and Sun [11] and Twilley et al. [31], who used fruity, floral, honey-like, alcoholic, vegetal, and chemical as odor descriptors for different types of mead. Li and Sun [11] noted that the honey type can significantly influence the aroma of mead. According to their study, mead manufactured from linden honey had a higher aroma quality and intensity in comparison to mead made of multiflorous honey. Samples of ‘T’ and ‘TS’ were also characterized as clove-like (2.0; 4-allyl-2-methoxyphenol) and sweet (1.0–1.5). Thereby, the sweet aroma was scored higher in ‘T’ (1.5) in comparison to ‘TS’ (1.0). A decrease in the sweetness note can be crucial because it plays an important role in consumer’s acceptance of mead [15]. Pereira et al. [33] reported that sweeter mead received higher scores during consumer tests. The appearance of a sweet aroma might be linked to the addition of some spices with a sweet note (e.g., vanilla-like, cinnamon-like). The aroma was also defined as fruity in both ‘T’ and ‘TS’ on the same level (0.5). The fruity aroma of ‘T’ and ‘TS’ was scored lower than in the study performed by Li and Sun [11] and also than in case of rum studied by Franitza et al. [20]. However, as mentioned by Li and Sun [11], the intensities of specific odors strictly depend on the type of yeast used during mead preparation, and the time of wort boiling plays an important role as well. In the present study, no off-flavor compounds that could be linked to long heat treatment with characteristic rubbery and resin-like smell [5] were formed in ‘T’ and ‘TS’.

## 3. Materials and Methods

### 3.1. Chemicals

The following compounds, used as authentic reference compounds for GC-O and HS-SPME-HRGC-MS, were commercially available: acetic acid, 2-acetylpyrazine, 4-allyl-2-methoxyphenol, 1,8-cineol, coumarin, γ-decalactone, dodecanoic acid, ethyl acetate, ethyl decanoate, ethyl hexanoate, ethyl-3-methylbutanoate, ethyl octanoate, ethyl-3-phenylpropanoate, hexanoic acid, 3-hydroxy-4,5-dimethyl-2(5*H*)-furanone (sotolon), 4-hydroxy-2,5-dimethyl-3(2*H*)-furanone (furaneol^®^), 3-methylbutyl acetate, methyl 3-(methylthio)propanoate, methyl propanoate, 2-methyl-1-propanol, 3-(methylthio)propionaldehyde (methional), octanal, octanoic acid, 1-pentanol, pentyl acetate, phenylacetic acid, 2-phenylethanol, and *trans*-whisky lactone (Sigma-Aldrich Chemie, Taufkirchen, Germany), diethyl succinate (Supelco, Bellefonte, PA, USA), 3-methylnonane-2,4-dione (Chemos, Regenstauf, Germany), and 4-hydroxy-3-methoxybenzaldehyde (vanillin) (Merck, Darmstadt, Germany).

The following stable isotopically labeled internal standards were commercially available: [^2^H_3_]-1,8-cineol, [^2^H_3_]-diethyl succinate, [^2^H_3_]-ethyl acetate, [^2^H_3_]-ethyl decanoate, [^2^H_3_]-ethyl hexanoate, [^2^H_9_]-ethyl 3-methylbutanoate, [^2^H_3_]-ethyl octanoate, [^2^H_2_]-2-methoxy-4-(1-propenyl)phenol, [^2^H_2_]-2-methylbutanal, [^2^H_3_]-2-methyl-1-propanol, [^13^C_2_]-phenylacetic acid, and [^2^H_5_]-2-phenylethanol (Sigma-Aldrich Chemie).

Liquid nitrogen was obtained from Linde (Munich, Germany). Diethyl ether and *n*-pentane (Merck) were freshly distilled prior to use, and hydrochloric acid and sodium carbonate were purchased from Merck. All chemicals were at least of analytical grade.

### 3.2. Preparation of Mead Samples

The experimental material included ‘trójniak’ type of mead (1:3, *v*:*v*, honey to water), with wort boiling ‘trójniak sycony’(TS) and without wort boiling ‘trójniak’ (T). Mead samples were purchased from a local brewer from the south of Poland. According to the producer’s instructions, the mead was prepared using multiflorous honey and *Saccharomyces cerevisiae* yeasts (SafSpirit HG-1, Fermentis Lesaffre for Beverages). During the fermentation process, the pH value of mead wort was controlled, and a pH value around 5.0 was determined as the final one. About 30 L of each model mead were prepared, and in a final step filtered to achieve clarified liquids that were bottled. Samples were stored at 4 °C in the dark until analysis.

### 3.3. Isolation of the Volatiles and Their Analysis by Gas Chromatography-Olfactometry/Flame Ionization Detection (GC-O/FID)

Mead (100 mL) was extracted with diethyl ether (3 × 100 mL) by vigorous shaking at room temperature. The combined organic phases were washed with an aqueous NaCl solution (1 mol/L; 3 × 300 mL), which was previously described as a method to remove most of the ethanol by Franitza et al. [20]. To separate the volatile fraction from non-volatiles, a high vacuum distillation using the solvent assisted flavor evaporation (SAFE) technique was applied [34]. The distillate obtained was concentrated using a Vigreux column (50 cm × 1 cm i.d.) to ~4.5 mL, followed by microdistillation to a final volume of ~200 µL.

For GC-O/FID, a TRACE GC 2000 (ThermoQuest, Egelsbach, Germany) equipped either with a DB-FFAP capillary column (30 m × 0.25 mm i.d., 0.25 µm film thickness) or with a DB-5 capillary column (30 m × 0.32 mm i.d., 0.25 µm film thickness; both J&W Scientific; Agilent Technologies, Waldbronn, Germany) was used. Aliquots of the samples (1 µL) were applied by the cold on-column technique. The oven temperature started at 40 °C, held for 2 min, then raised at a rate of 6 °C/min to 230 °C, and again held for 5 min. Helium was used as the carrier gas with a flow rate of 2.0 mL/min. At the end of the column, the effluent was split 1:1 by a Y-type quick-seal glass splitter (Chrompack, Frankfurt, Germany) and one part was directed to an FID held at 250 °C, and the second one to a sniffing port held at 230 °C. Linear retention indices (RIs) were calculated for each compound using a mixture of *n*-alkanes (C_6_-C_26_ for DB-FFAP and C_6_-C_18_ for DB-5, respectively).

### 3.4. High-Resolution Gas Chromatography-Mass Spectrometry (HRGC-MS)

HRGC-MS was performed by a Hewlett-Packard gas chromatograph 5890 series II (Agilent Technologies) coupled to a Finnigan sector field mass spectrometer type MAT 95S (Bremen, Germany). The same DB-FFAP and DB-5 capillary columns mentioned above were used for this measurement. Mass spectra were generated both in electron ionization (EI) mode at 70 eV and in chemical ionization (CI) mode at 115 eV using isobutane as reagent gas.

### 3.5. Determination of Mead Volatiles by Headspace-Solid Phase Microextraction-High-Resolution Gas Chromatography-Mass Spectrometry (HS-SPME-HRGC-MS)

The volatile compounds were isolated by HS-SPME and analyzed by a Varian 3800 gas chromatograph (Darmstadt) equipped with a DB-FFAP capillary column (30 m × 0.25 mm i.d., 0.25 µm film thickness; J&W Scientific) coupled to an ion trap mass spectrometer Saturn 2000 (Varian) running in CI mode at 70 eV, with methanol as the reagent gas. Sample injections were performed by a CombiPAL autosampler (CTC Analytics, Zwingen, Switzerland) and a CAR/PDMS fiber (Supelco) was used for all experiments. The SPME conditions were applied according to a description of Senn et al. [35] with minor modifications. Therefore, sampling was performed for 30 min at 40 °C, and NaCl (1 g) was added to mead (5 mL) and weighed into gas-tight sample vials (20 mL). All analyses were performed in triplicates.

### 3.6. Descriptive Sensory Analysis of Mead Samples—Aroma Profile Analysis (APA)

For APA, the intensities of five selected odor descriptors (ethanolic (represented by an aqueous ethanolic solution), fruity (ethyl hexanoate), clove-like (4-allyl-2-methoxyphenol), sweet (vanillin), and honey-like (phenylacetic acid)) were rated on a seven-point linear scale from 0 to 3 by steps of 0.5 (from 0 = not perceivable to 3 = strongly perceivable). APA was performed according to Zhai and Granvogl [36]. The panel consisted of 15 experienced assessors, who participated in weekly training for the recognition and description of aroma attributes. The samples were presented in covered odorless Teflon^®^ vessels in a sensory room equipped with individual booths at 21 °C.

### 3.7. Statistical Analysis

The data are presented as mean values of triplicates and corresponding standard deviations. The differences between the samples were analyzed by a one-way ANOVA with Tukey’s multiple comparison test (*p* < 0.05) using STATISTICA 13.1 (StatSoft Inc., Tulsa, OK, USA).

## 4. Conclusions

By applying the molecular sensory science concept to two different types of mead (with and without wort boiling), 28 aroma-active compounds were identified. After quantitation via SIDAs using HS-SPME-HRGC-MS, ethyl acetate, ethyl hexanoate, 1-pentanol, 2-phenylethanol, phenylacetic acid, and 2-methyl-1-propanol were identified as most abundant volatiles in mead. However, by considering the orthonasal odor thresholds and subsequent OAV calculation, the esters ethyl hexanoate, ethyl octanoate, and ethyl 3-methylbutanoate, all with fruity odor notes, were proven to be the most important aroma-active compounds in both mead samples. Moreover, results obtained by AEDA might suggest 2-acetylpyrazine as a characteristic volatile mead compound formed during wort boiling. The relationship between overall mead volatile composition and sensory evaluation should be further investigated to verify the key volatiles by means of omission or recombination experiments. Finally, the controlled boiling of wort for mead production can lead to products with high aroma quality.

## Figures and Tables

**Table 2:** Selected ions (*m*/*z*) of analytes and stable isotopically labeled internal standards and response factors (R_f_) used in stable isotope dilution assays (SIDAs).

Compound	Isotope Label	Ions (*m*/*z*) ^1^		Rf ^2^
		Analyte	Internal Standard	
4-allyl-2-methoxyphenol ^3^	[^2^H_2_] ^3^	165	167 ^3^	0.80
1,8-cineol	[^2^H_3_]	155	158	0.87
diethyl succinate	[^2^H_3_]	175	178	0.77
ethyl acetate	[^2^H_3_]	89	92	0.95
ethyl decanoate	[^2^H_3_]	201	204	0.96
ethyl hexanoate	[^2^H_3_]	145	148	0.98
ethyl 3-methylbutanoate	[^2^H_9_]	131	140	1.00
ethyl octanoate	[^2^H_3_]	173	176	0.98
2-methyl-1-propanol	[^2^H_3_]	75	78	0.89
1-pentanol ^4^	[^2^H_2_] ^4^	89	89 ^4^	1.00
phenylacetic acid	[^13^C_2_]	137	139	0.90
2-phenylethanol	[^2^H_5_]	105	110	0.71

^1^ Ions used for quantitation in chemical ionization mode. ^2^ Response factors determined by analyzing defined mixtures of unlabeled analyte and corresponding stable isotopically labeled internal standard. ^3^ 4-Allyl-2-methoxyphenol was quantitated using [^2^H_2_]-2-methoxy-4-(1-propenyl)phenol as the internal standard. ^4^ 1-Pentanol was quantitated using [^2^H_2_]-2-methylbutanal as the internal standard.

**Table 3:** Concentrations of aroma-active compounds of mead ‘trójniak’ (T) and ‘trójniak sycony’ (TS) determined by SIDAs *via* HS-SPME-HRGC-MS analysis.

	Concentrations ^1^ [µg L^−1^]	
Compound	T	TS
ethyl acetate	16,400 ^b^	57,000 ^a^
ethyl hexanoate	1220 ^a^	1230 ^a^
1-pentanol	980 ^a^	966 ^a^
2-phenylethanol	820 ^a^	551 ^b^
phenylacetic acid	748 ^a^	770 ^a^
2-methyl-1-propanol	695 ^b^	1050 ^a^
ethyl decanoate	610 ^a^	612 ^a^
diethyl succinate	539 ^a^	536 ^a^
ethyl octanoate	405 ^a^	408 ^a^
4-allyl-2-methoxyphenol	300 ^b^	560 ^a^
ethyl 3-methylbutanoate	250 ^a^	160 ^b^
1,8-cineol	90.2 ^b^	150 ^a^
Total	23,007	63,958

^1^ Mean values of triplicates with standard deviations ≤ 10%. ^a,b^ Mean values with different letters in the same row are statistically different (*p* < 0.05; Tukey’s test).

**Table 4:** Odor thresholds (OTs) and odor activity values (OAVs) of important aroma-active compounds of mead ‘trójniak’ (T) and ‘trójniak sycony’ (TS).

Compound	OT ^2^ [µg L^−1^]	OAVs ^1^	
		T	TS
ethyl hexanoate	4	305	306
ethyl octanoate	1.6	253	255
ethyl 3-methylbutanoate	1.6	156	100
1,8-cineol	3.2	28	46
2-methyl-1-propanol	50	14	21
4-allyl-2-methoxyphenol	50	6	11
ethyl decanoate	244	3	3
ethyl acetate	7500	2	8
phenylacetic acid	6100	<1	<1
2-phenylethanol	7500	<1	<1
1-pentanol	30000	<1	<1
diethyl succinate	300000	<1	<1

^1^ Odor activity value was calculated as the ratio of the concentration (cf. Table 3) to the respective orthonasal odor threshold. ^2^ Orthonasal odor threshold was previously reported in ethanol/water (9/91, *v*/*v*) [14].

## Data Availability

The data presented in this study are available on request from the corresponding author.

## Sample Availability

Samples of the compounds are available from the corresponding author.
